# User-centred design of a clinical decision support system for palliative care: Insights from healthcare professionals

**DOI:** 10.1177/20552076221150735

**Published:** 2023-01-10

**Authors:** Vicent Blanes-Selva, Sabina Asensio-Cuesta, Ascensión Doñate-Martínez, Felipe Pereira Mesquita, Juan M. García-Gómez

**Affiliations:** 1 Biomedical Data Science Lab, Instituto Universitarios de Tecnologías de La Información y Comunicaciones (ITACA), 16774 Universitat Politècnica de València, Valencia, Spain; 2 Polibienestar Research Institute, 16781 University of Valencia, Valencia, Spain; 3 Divisão de Hematologia, 28113 departamento de Clínica Médica, da Universidade Federal de Juiz de Fora, Minas Gerais, Brasil

**Keywords:** CDSS, design, machine learning, palliative care, user-centred validation, usability

## Abstract

**Objective:**

Although clinical decision support systems (CDSS) have many benefits for clinical practice, they also have several barriers to their acceptance by professionals. Our objective in this study was to design and validate The *Aleph* palliative care (PC) CDSS through a user-centred method, considering the predictions of the artificial intelligence (AI) core, usability and user experience (UX).

**Methods:**

We performed two rounds of individual evaluation sessions with potential users. Each session included a model evaluation, a task test and a usability and UX assessment.

**Results:**

The machine learning (ML) predictive models outperformed the participants in the three predictive tasks. System Usability Scale (SUS) reported 62.7  ±  14.1 and 65  ±  26.2 on a 100-point rating scale for both rounds, respectively, while User Experience Questionnaire – Short Version (UEQ-S) scores were 1.42 and 1.5 on the −3 to 3 scale.

**Conclusions:**

The think-aloud method and including the UX dimension helped us to identify most of the workflow implementation issues. The system has good UX hedonic qualities; participants were interested in the tool and responded positively to it. Performance regarding usability was modest but acceptable.

## Introduction

Clinical decision support systems (CDSS) are computer systems designed to assist clinicians in their decisions on individual patients when these decisions are actually being made.^1^ Interest and research in CDSS are motivated by their potential benefits, which have been documented in the scientific literature: increased patient safety by reducing medical errors or avoiding advice against protocols; improved service quality due to better adherence to guidelines, and increased service time dedicated directly to the patients; cost reduction by faster processing of the demands and avoiding duplicated tests; improved administrative functions by incorporating elements such as automatic documentation; and diagnosis support and workflow improvement.^2,3^

However, despite CDSS’ multiple virtues, these systems have not been widely adopted in clinical practice.^4,5,6,7,8^ Several studies have pointed out the main barriers to their adoption, divided into two broad categories: sociocultural factors and usability. The former refers to the beliefs of healthcare professionals (HCPs) or their organization regarding the CDSS, such as the idea of a loss of autonomy, the feeling of being replaced by the system, low computer literacy, lack of trust in the system, failure to fulfil a perceived clinical need, legal uncertainties and a misalignment between human needs and the technical system.^9,10,11,12^ Liberati et al.^10^ proposed several strategies to deal with these barriers according to the physicians’ beliefs about these systems, which are mostly based on communication, training, and highlighting the system's benefits.

Usability barriers refer to the users’ difficulties with the associated software. The most common problems in this category are the difficulty of operating the software, disruption of the workflow, loss of face-to-face time with patients^2^ and the alert fatigue due to excessive notifications by the system.^12,13^ These challenges have previously been addressed by other authors through usability pilots with the potential software end users, mostly HCPs, to identify and correct the different CDSS usability problems.^13,14,15,16^

Usability studies often follow a general scheme. The participants are exposed to the software in a controlled environment and the session is taped and/or with the researchers taking field notes. The participants must try to accomplish tasks in actual scenarios, which in some studies receive the name of ‘near-live’ simulations.^17^ The think-aloud method^18^ is commonly used during the whole study. This method consists of asking the participants to express their doubts, opinions and in general any of their thoughts on their experience with the tool. The software usability is finally quantified through a scale or an index, one of the most popular evaluation tools being the System Usability Scale (SUS).^19,20^

It is generally accepted that a positive user experience (UX) is essential to any software acceptance.^21^ Despite the close relationship between usability and UX concepts, there are some differences worth studying, primarily related to the hedonic category,^22^ that is, how ‘pleasurable’ the users find it to use the software. UX also studies emotions, beliefs, preferences and perceptions. These concepts directly impact the adoption of a CDSS because they are closely related to the previously mentioned sociocultural barriers. A UX study is thus essential to assess and improve the adoption of the technology.

Another crucial aspect of maximizing the probability of a successful CDSS implementation in clinical practice is the initial design. An interdisciplinary team is highly recommended, including data scientists, programmers, usability and UX experts, HCPs as potential users of the software and other stakeholders such as representatives of hospital management to obtain a clear vision of the requirements.^11,23^ Planning a pleasant interface is also important since some studies have reported users being more tolerant of minor usability issues if they found the interface visually appealing. This is known as the *aesthetic-usability effect*.^24^

CDSSs are in a very early stage of development in palliative medicine. The study by Tan et al.^25^ presents an example of a CDSS specifically developed for PC, designing and implementing a CDS tool aimed at identifying patients in emergency departments that could benefit from primary PC, which demonstrated a very positive usability value. In the context of PC, several tools are designed to support HCPs in detecting patients in need of PC (Maas et al.),^26^ but this is still one of the challenges dealt with by HCPs in their clinical routines, especially when they treat patients with non-malignant conditions.^27,28^ In this line of research, we developed a set of predictive models to assist palliative care (PC) referrals with hospital admission data on older patients using mortality and frailty predictions as the main criteria.^29^ The result of that study was a demonstrator for a complete CDSS known as The *Aleph PC*. Our study reported that these models accurately identified patients with a short survival time who were likely to become frail. Our goal in the present work is to validate The Aleph PC through user-centred techniques^30^ to determine how different HCPs with PC experience envision using a PC CDSS in their clinical practice. First, we evaluated The Aleph PC's mortality and frailty models against HCPs’ predictions to obtain a baseline and then we assessed the usability and UX of the system alongside the different insights of the HCPs on how to build a useful PC CDSS.

## Materials and methods

### The Aleph CDSS platform

**Figure 1.**
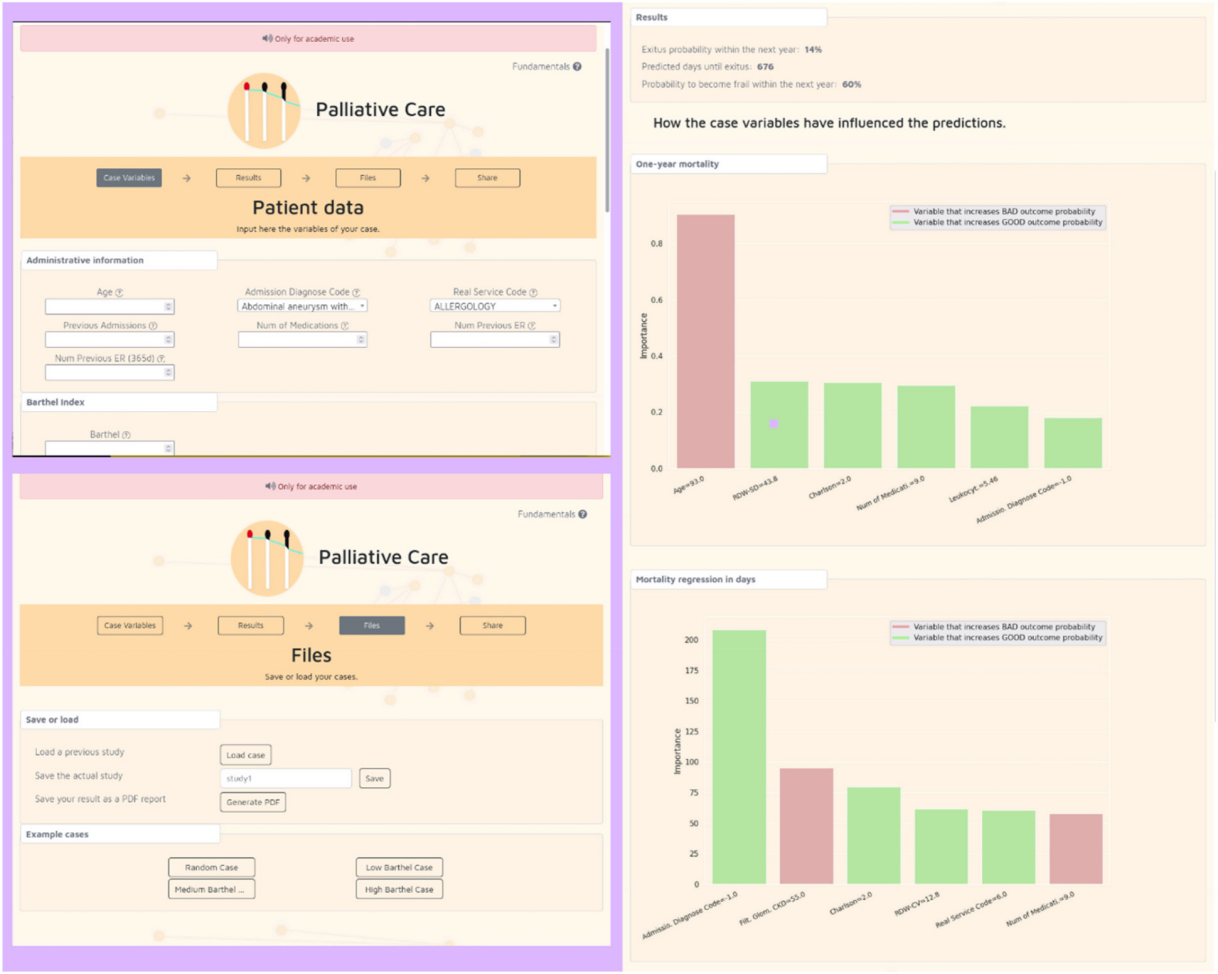
(a) (left-top) Screen in which the user inputs the data, (b) (right) screen showing the results and (c) (left-bottom) screen for managing files with predetermined examples.

The Aleph PC is an open-access machine learning (ML)-based CDSS implemented as a web platform. The application is divided into three main screens: the user introduces the different data required for the PC predictions in the first, including administrative information, Barthel^31^ and Charlson^32^ indexes, laboratory results and a few diagnostic variables (Figure 1(a)). After completing the form, the results are calculated and displayed on another screen (Figure 1(b)). These results include a numerical result for each model and an ML explainability figure. We used the Shapley value (SHAP)^33^ to display a graph with the relation between the input and the prediction obtained. The files section (Figure 1(c)) allows the user to save the current case, load a different case or test the application with predefined test cases. The version tested in this study can be accessed here: https://demoiapc.upv.es/ (last accessed 29 June 2022).

### Recruitment process

HCPs involved in different aspects of treating patients with bad prognoses and possible end CDSS users were recruited for the study. We focused especially on nurses, primary care physicians, hospitalist physicians, PC consultants and specialists like oncologists, neurologists or pulmonologists. This decision ensured including the different approaches to working with complex patients in need of PC. By means of the Snowball sampling technique,^34^ the authors drafted a list of possible participants with not directly connected with the development of The Aleph PC, including clinical partners from the InAdvance Project and other relevant institutions that actively participated in research in the PC field from six different countries: Italy, Brazil, Spain, Greece, Scotland and Portugal. The first volunteers were asked to suggest other colleagues willing to participate until we completed our target sample size. The invitations to participate in the study were sent by email.

### Study structure

#### Participation

The study was defined as an iterative user-centred validation. The participants were invited to individual evaluation sessions in which a team member acted as the session guide. Due to the reactions of the first participants, who asked whether the sessions would be recorded when they were first contacted, it was decided not to record the sessions. We understood this hesitance to be recorded as a possible barrier to recruitment, especially with volunteers that do not belong to our organizations who gave us their valuable time for free, and there is normally no need to review a user test on video since one is mostly interested in finding the major ‘usability catastrophes’.^35^ The team was instructed to record the users’ general attitude as well as their reactions, including verbatim quotes that could help to document the ideas discussed in the session. A second member of the team was occasionally present at the evaluation session to take field notes.

**Figure 2.**
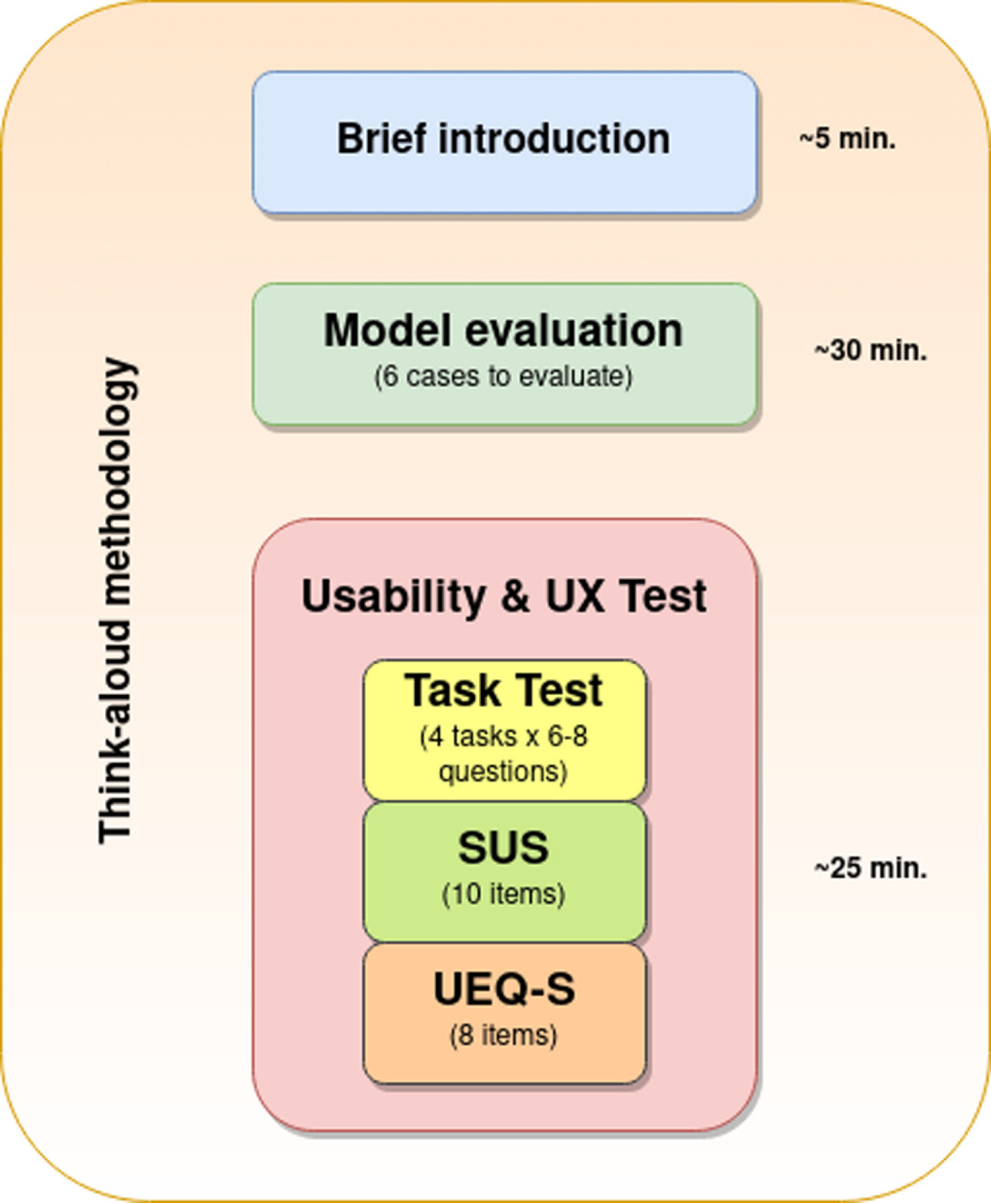
Overview of the structure of the sessions, including three sections and the approximate time spent on each: (1) Brief introduction, (2) Model evaluation and (3) Usability and user experience (UX) test.

The evaluation sessions were by videoconference, in which the participants shared their screens while interacting with Aleph PC Using the think-aloud method.^17^ Each session lasted around 1 hour. Their overall structure is shown in Figure 2.

We defined two rounds of sessions, separated by a period of 15 days, in which we evaluated the participants’ comments during the sessions, followed by a consensus on how to address each issue and how to adapt the software according to the feedback received. Sixteen participants were invited to the first round and eight different participants to the second. We aimed to carry out more sessions in the first round to detect as many usability problems as possible so we could compare the number and the nature of the identified issues.

#### Model validation

The model was first evaluated in an unlinked section of The Aleph PC. After introducing some basic information, the participants saw six vignettes consisting of already filled-in, non-editable input forms of actual cases. They were then asked to fill in their own 1-year mortality (1ym) predictions (yes/no), mortality regression (months of interval) and 1-year frailty. All 21 participants were questioned about the same vignettes in the same order in both rounds. There was no time limit and the participants were asked to use any information resource (internet search, books, etc.) they needed to make their own predictions.

The accuracy, sensitivity and specificity^36^ of the 1ym and the 1-year frailty (Frailty) models were calculated. Since we asked the participants for their predictions in months to facilitate their responses, we had to transform the output of the regression model from days to months, and so we divided the number of days by 30. As the results of our previous study had found an interval of 4 months, we used the prediction in months  ±  2 months as the interval bounds. We then calculated the accuracy of the participants and the model by checking whether the real value of the cases in months belonged to the interval (lower bound < = real value < = higher value).

#### Usability and UX validation

In the usability and UX section, the participants answered a Google Forms questionnaire (last accessed 21September 2022; shorturl.at/apV23) while they were testing The Aleph PC in a ‘task test’ consisting of four simple tasks and answering a series of questions after each one. The tasks covered all the implemented functionality of the CDSS: (1) input a feasible case, (2) check the results and interpret the graphics, (3) save the current case and (4) load a previously stored case. The questions after each task covered any difficulties experienced, the perception of time spent, the number of errors found by the participant (including unexpected behaviours and any elements they did not understand) and the level of satisfaction obtained by performing the task. All the questions were mandatory.

After the task test, the usability and experience were tested by the SUS questionnaire^19,20^ and the User Experience Questionnaire – Short Version (UEQ-S)^37^ both implemented on the same Google Forms page. The participants were asked to stop sharing their screens upon the completion of both tests.

## Results

### Participation

Fifteen of the 16 initial participants agreed to participate (93.75%) and 6 of the 8 initially invited to the second round responded positively (75%). We settled on six respondents for the second round, due to the difficulty of finding participants and the fact that, according to Nielsen,^38^ we already had enough participants to detect most of the usability problems.

The distribution of the participants in both rounds was the following: 15 were physicians with the following roles: 7 general practitioners, 5 hospitalists, 1 PC consultant, 1 oncologist and 1 neurologist. The other 6 were nurses. The distribution between sex was 13 males (61.9%) and 8 females (38.1%), of which 5 were Italian, 4 Brazilian, 4 Spanish, 4 Greek, 2 Scottish and 2 Portuguese.

### Model evaluation

The ML models outperformed the HCPs’ predictions in both mortality and frailty (Table 1). The mean width of the intervals provided by the participants in the regression prediction was: a 16.2-month 95% confidence interval (CI) (13.5 to 18.9) against the models’ fixed 4 months.

**Table 1.** Summary of the metrics for the participants and the machine learning (ML) models in the three tasks for the six cases evaluated. Mean and 95% confidence intervals (CIs) are reported per participant. ML is deterministic so no variability in the prediction was found.

Task	Predictions	Accuracy	Sensitivity	Specificity
1ym	Participants	0.5 [0.42, 0.58]	0.54 [0.42, 0.56]	0.46 [0.34, 0.58]
	The Aleph PC	0.83	0.75	1
Frailty	Participants	0.78 [0.7, 0.85]	0.8 [0.72, 0.88]	0.67 [0.45, 0.89]
	The Aleph PC	1	1	1
Regression	Participants	0.45 [0.36, 0.55]	-	-
	The Aleph PC	0.67	-	-

PC: palliative care; 1ym: 1-year mortality.

### Qualitative results

A list of improvements was compiled after each round regarding the qualitative results based on the think-aloud method and the authors’ notes on the participants’ behaviour. The changes were focused on interface details: removal of the diagnosis-related group variable because it could be inferred from the International Classification of Diseases 9th revision (ICD9) code, replacement of the ICD9 codes by their name, improved tooltip descriptions and added reference values for the laboratory variables. Supplementary Table 2 contains the complete log of changes introduced in both rounds. Most of the participants provided feedback on the subset of variables, suggesting other variables they were more familiar with or discarding the existing variables as unimportant or unavailable in their workflow (e.g. ‘Nurses in Portugal don't use lab results, they understand them but they don't work with them’, nurse first round). The feeling towards the CDSS was primarily positive, and the idea of PC identification by ML technology was well received (e.g. ‘I think that having a tool like this in my day-to-day work could help to manage the patients’, nurse second round). Few of the participants felt confused on their first interaction with the software but many said they had learned to use it after the tasks test (e.g. ‘I found the platform easy to use but some inconsistencies need to be improved’, Physician, first round). Participants from hospital settings suggested automatically collecting the diagnosis and laboratory results from the electronic health records (EHR) (e.g. ‘Different labs may have different measurement units, so it will be important to specify them. Also, it would be better if the application could read this information from the EHR’, physician first round), while other participants did not care about complete integration due to the lack of system integration in their respective environments. We found a participant in each round who was sceptical about the use of computers for decision-making and did not believe in the benefits of the technology, rating every aspect of the system very low in the different questionnaires and providing poor opinions of the system through the think-aloud method (e.g. ‘I won’t use a tool like this in my practice. These predictions are useless for me’, physician second round).

### Performance of task

**Figure 3.**
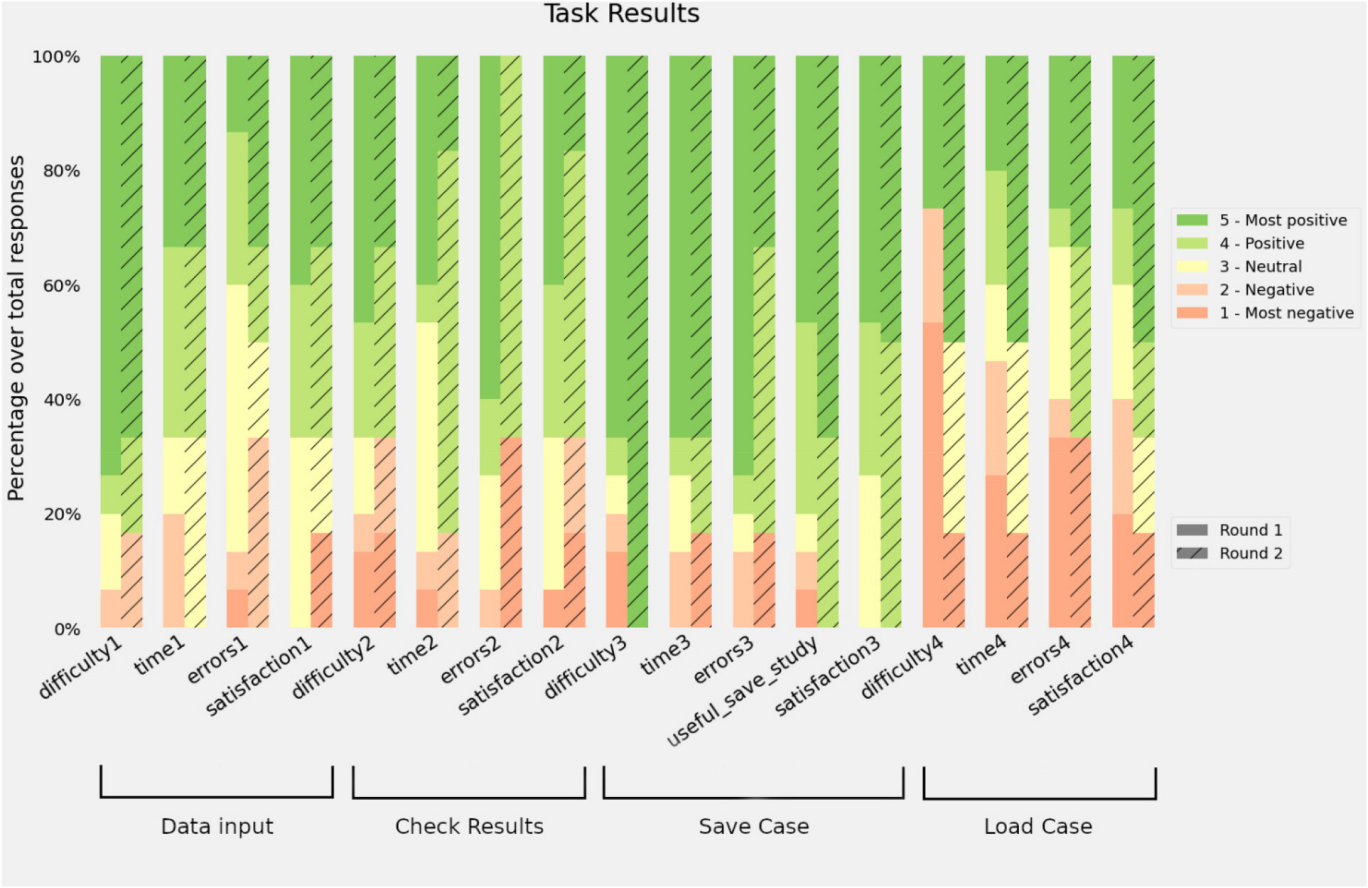
Results of the different tasks during the task test. Bars represent the distribution of the responses. A positive response means that the participant found the task: easy, short, with few errors or satisfactory.

Figure 3 shows the distribution of the answers in both rounds. Almost every measured feature increased the percentage of positive feedback during the second round. The most significant improvement was in task four (load a case), in which the perceived difficulty, time spent and satisfaction improved despite the greater number of errors.

### Usability

**Figure 4.**
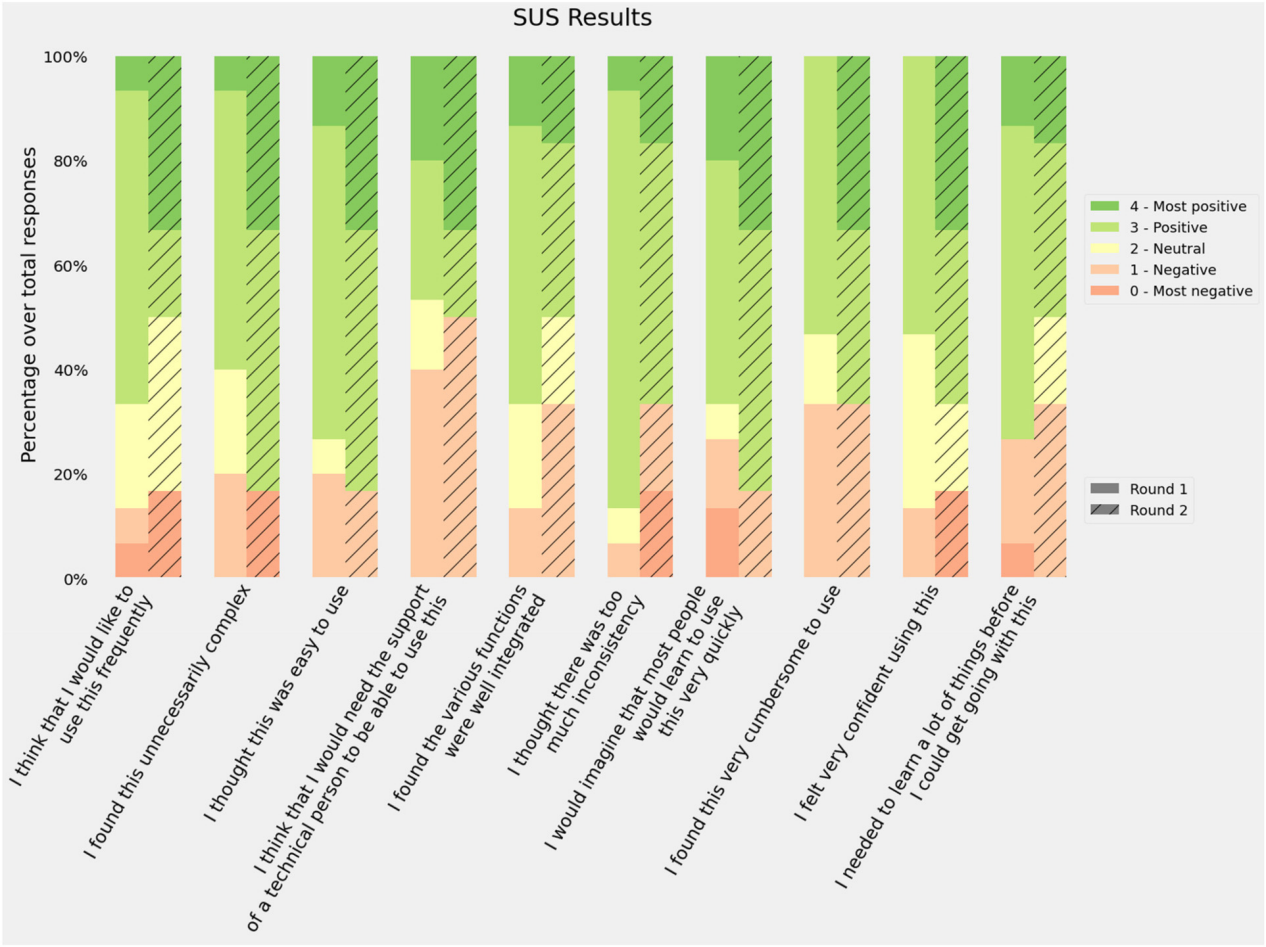
Responses to the System Usability Scale (SUS) questionnaire. Bars represent the distribution of the responses using the adjusted scores: raw scores −1 for items in the odd position and raw scores −5 for items in an even position.

Responses to the 10 SUS item scores were recorded, all items were mandatory so no missing values were present. The first round of the evaluation sessions obtained a mean of 62.7  ±  14.1 while the second round increased its score to 65  ±  26.2. The distribution of the answers for the different items is shown in Figure 4. The adjusted scores were used instead of the raw scores for all items to help with visualization. Round 2 had more positive responses in 6 of the 10 items: ‘I found this unnecessarily complex’, ‘I thought this was easy to use’, ‘I think that I would need the support of a technician to be able to use this’, ‘I would imagine that most people would learn to use this very quickly’, ‘I found this very cumbersome to use’ and ‘I felt very confident using this’. However, the first round obtained a lower score and SD.

Previous studies have tried to map the intervals of the score into categories such as ‘Poor’, ‘OK’ or ‘Good’ or school grading scales^39^ in order to provide a better usability reference. According to these frameworks, our results for both rounds would be classified as D (lowest passing score), the first round as ‘OK - low marginal acceptance’ and the second as ‘OK - high marginal acceptance’. However, if we recalculate the SUS average score excluding the sceptical participants, the average rating would be 63.9  ±  13.8 and 74.5  ±  16.8, which are D ‘OK - High marginal acceptance’ and C ‘Good - Acceptable’.

### UX

**Figure 5.**
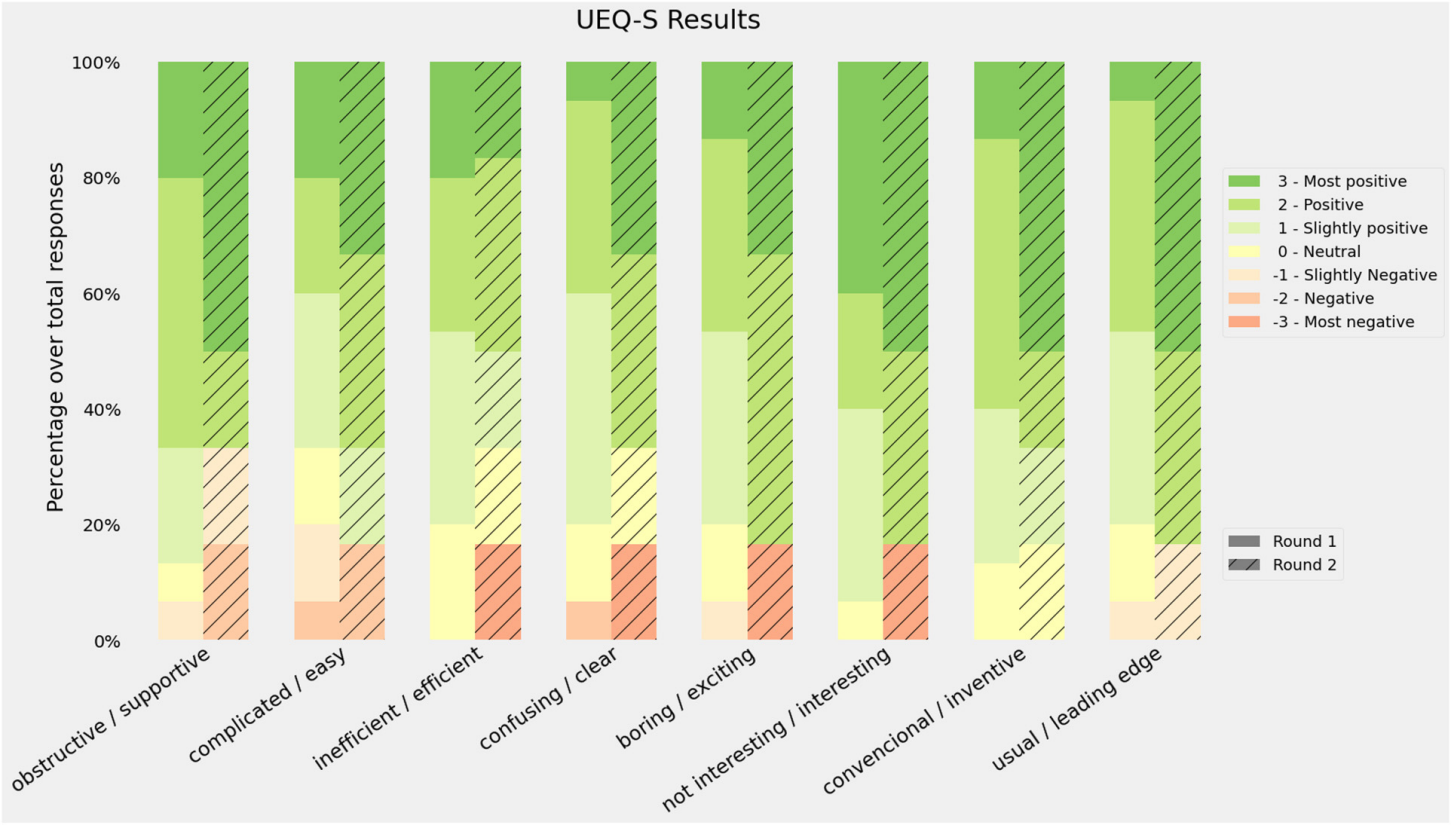
Results of the User Experience Questionnaire (UEQ). Bars represent the distribution of the response. Positive responses mean that the participants agreed with the positive quality of the software (supportive, easy, etc.).

Answers to the UEQ-S questionnaire were recorded, with all items being mandatory. Figure 5 shows the distribution of the responses for each item in the questionnaire. The median for the second round was always greater than the first round, and the average scores were 1.4 in the first round and 1.5 in the second. The Pragmatic score was slightly higher in the first round (1.3 vs. 1.2) and the hedonic score improved during the second round (1.5 vs. 1.8).

**Figure 6.**
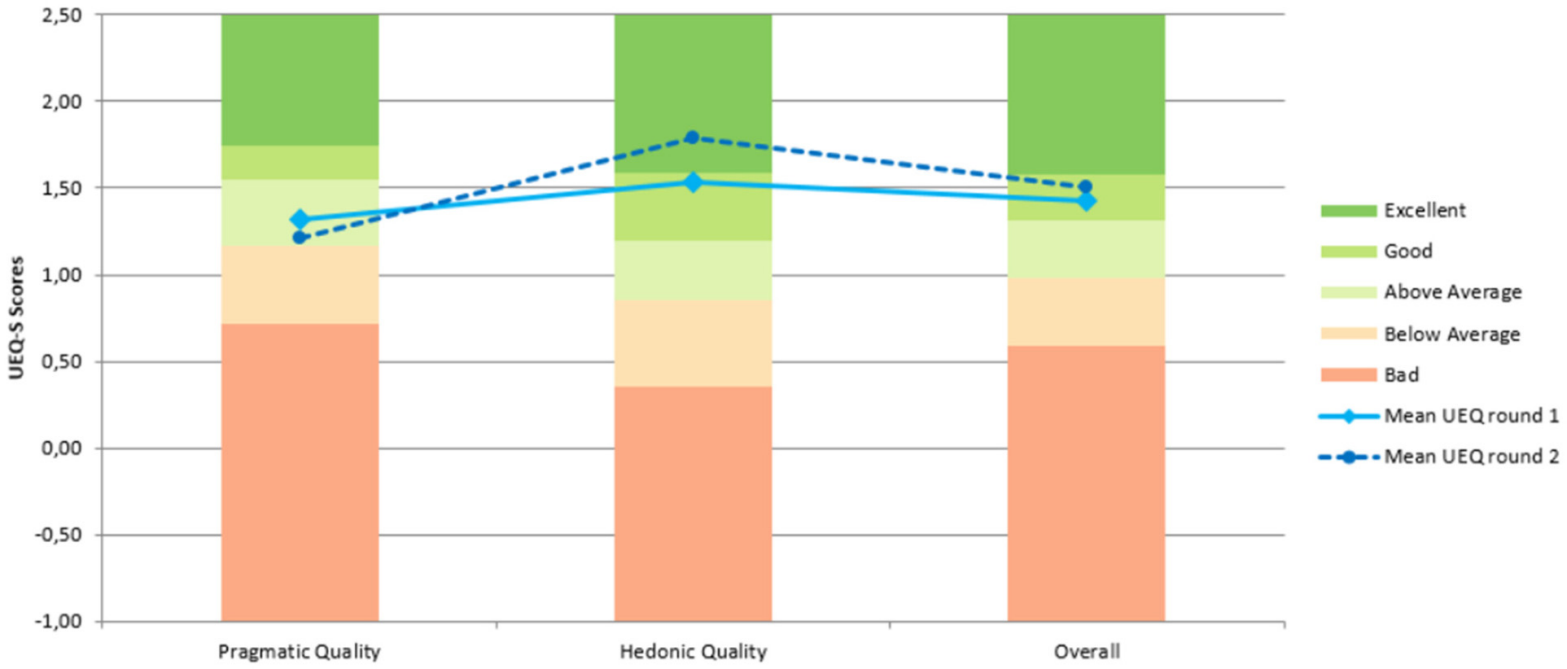
User Experience Questionnaire (UEQ) results by categories against the official benchmark. The grade assigned to each category depends on the results of the different studies used to create the benchmark.

The authors of the UEQ-S provide a benchmark to compare the study results. Although this benchmark is intended for the full-size UEQ, the results may be acceptable for estimating UX. Figure 6 shows the results of both rounds in the three categories and their benchmark score.

## Discussion

This study took the form of an iterative user-centred validation of a CDSS aimed to support HCPs in identifying patients in need of PC. The two-round validation process involved decision, usability and UX tests. The predictions provided by the models were found to be more accurate in both sensitivity and specificity metrics for both classification models than those provided by the participants. The regression model accuracy result depended on the width of the interval; we selected 4 months as an acceptable error based on the original reported mean absolute error.^29^ Other studies have described clinicians’ poor accuracy when predicting 1ym through mechanisms like the Surprise Question (‘Would I be surprised if this patient died in the next 12 months?’).^40^ However, our intention with this comparison was to set a reasonable baseline for the predictive models. There are several factors that work against clinicians’ performance in this evaluation: (1) clinicians are not good at taking decisions based only on EHR data,^41^ (2) not having physical access to the patient affects HCPs’ intuition^42^ and (3) few cases were evaluated. However, these results indicate that The Aleph PC could help improve the clinical predictions with data.

The results of the task test indicate that the four tasks were not perceived as difficult. Task 4 (load a file in the platform) presented the highest number of negative responses for its difficulty in the first round, but this improved in the second round after we relocated the load button from the input variables section to the files section, as suggested by the participants. The tasks were also perceived as easy to carry out, despite the need to input all the variables manually in the first task. Levels of satisfaction were high, increasing in the last two tasks in the second round after the interface improvement. It is worth mentioning that all the participants in the second round rated the option to store the data in a spreadsheet file positively as they would be able to revisit the case later.

The different SUS dimensions were considered positive (scores 3 and 4) by at least half of the participants in both rounds. The best scores were related to the perceived difficulty: ‘I found the system unnecessarily complex’, ‘I thought the system was easy to use’ and ‘I would imagine that most people would learn to use this system very quickly’. Consistency was also rated among the best (‘I thought there was too much inconsistency in this system’). The worst scores were related to the participants’ confidence (‘I found the system very cumbersome to use’, ‘I felt very confident using the system’ and ‘I think that I would need the support of a technician to be able to use this system’). These results suggest that participants found The Aleph PC easy to use but thought it contained some elements they were not familiar with and/or needed further explanation. The average score was ‘passing’ though there is room for improvement according to participants’ feedback.

The UEQ-S results reported in Figure 5 provide two main interpretations, first, the median UX scores were higher in the second round after the introduction of improvements. Second, most of the dimensions kept their neutral (0) and positive (1, 2, 3) values. Some outlying responses were found, especially in the second round, from the sceptical participants, which mean that their experience with PC CDSS was positive. A further in-depth look into the supportive category is needed to address possible undetected issues.

After analysing the results from the SUS, the UEQ-S and the comments obtained from the think-aloud method, we obtained a wide variety of perceptions of the application. It seems that most of the participants were ready to use a tool like The Aleph PC to obtain prognostic predictions that could influence PC. Even the two participants who expressed their dislike for the CDSS and were sceptical about the tests had their own ideas of how the application should be: while the first-round participants did not feel that the tool was useful, the second-round participants specified ‘*The tool is boring. The end-of-life idea should be obtained in 10-15 seconds’*. Only a few participants had experience of using CDSS, but all of them understood these kinds of applications as supporting tools instead of as a threat to their autonomy^10,43,44^ and all of them understood the difficulty of identifying early PC patients, so that they were ready to accept a tool that could help them.

Although the usability results were not as positive as the hedonic quality. Some hypotheses could be extracted from the sessions. Mainly, The Aleph PC did not fit perfectly in its current status to the different participants’ backgrounds. Those from non-hospital environments commented on how some of the variables required by the models did not match with the information managed by their centres. Being unfamiliar with the required inputs could have a detrimental effect on its perceived ease of use. Four of the participants commented on the nature of the data introduced, they considered that introducing historical data in the application instead of a ‘snapshot’ of a given moment could be better predicting. Also, a couple of hospital physicians made suggestions about automating the input and the output and integrating them with the EHR, following the schema of integrative CDSS.^11^ These findings are in line with the concept of unremarkable computing,^41,45^ in which AI systems are meant to be integrated into the current workflow and not to disturb or overwork HCPs.

As stated in other works on validation, usability is a key factor in the success of CDSS implementation. Usability tests based on task performance are sometimes described as ‘near-live’ simulations, and the subsequent usability assessment is the standard used to discover usability issues and improve the final product.^13,46^ Nonetheless, developing a perfectly usable application does not guarantee its practical success, since there is a list of sociocultural barriers to adopting these technologies^10^ which are directly related to physicians’ vision and opinions and their organizations regarding these products.

Including the UX test in the evaluation sessions allowed us to detect the participants’ acceptance of Aleph and the general idea of using a CDSS in daily clinical routines. Despite the participants’ diversity of backgrounds, they agreed on the tool's usefulness. Also, with two exceptions, the whole set of participants believed in the technology and the evidence behind the predictive models. This is especially relevant since trust has been detected as one of the main issues in CDSS acceptance.^10^ Based on our sample, we could argue that a CDSS able to deal with PC decisions will probably overcome the social barrier of HCPs’ interest in the technology, although there are still crucial factors to be solved, such as the interest in the CDSS by the organization and the HPs’ trust in the systems’ predictions.

Including two rounds meant we could test whether the changes implemented after the first set of sessions influenced usability and UX in the second round. The difference between the overall SUS and UEQ-S scores were not found to be significant by the *t*-test (*p* > 0.05). However, we did find an improvement in certain dimensions of the metrics. We could not extract valid comparisons per role due to the sample size restrictions, since most of the nurses took part in the second round, and most of the physicians in the first round. However, these were not homogeneous groups and contained HCPs working in hospitals, primary care centres, external services and rural environments. We could have set more iteration to ensure that the minimum number of issues was kept in the software. However, the changes identified during the second round were either details such as the use of abbreviations and acronyms or barriers derived from the data source from which the models were created. We, therefore, considered that most of the fixable issues were identified and that we did not need to perform any extra iteration.

The participation of professionals with different roles and backgrounds showed us the diverse needs in highly heterogeneous PC implementation and workflows. There are significant differences between inpatient and outpatient settings,^47^ medical specialities^48,49,50^ and urban or rural environments.^51^ In these different contexts, physicians and nurses play different roles in identifying PC needs. For example, in the work by Zemplènyi et al.,^52^ the authors describe how nurses are the first to detect the needs and then the cases are discussed with the physicians. This may be different in other settings, such as rural areas, where physicians visit older patients. In our study, we found that participants working in non-hospital environments were more concerned about the availability of the variables, especially the laboratory results. Despite the possible barriers to using the tool in its current version, physicians and nurses thought they could benefit from a PC CDSS such as Aleph to identify patients in need of PC.

Including ML explainability in the system was another relevant detail in our implementation (Figure 1(b)). This could be defined as the human quality of understanding the relationship between the system input and the predictions^53^ and has been proposed many times as a solution to one of the most common CDSS adoption barriers, as stated by Shortliffe and Sepúlveda ‘black boxes are unacceptable’.^54^ CDSSs should be transparent to the user to allow them to accept or dismiss the prediction or recommendation. However, recent studies have highlighted possible problems when trying to create explainability mechanisms for single predictions. In their view, Ghassemi et al.^55^ discourage their implementation as patient-level systems. Since we received a positive feedback from the participants on this feature, we decided not to remove the explainability graphs after the second round. However, we do recognize the need for further study of these types of features and their impact on the clinical workflow.

Throughout this project, our team has followed the design recommendations of previous studies,^10,12,13,14,15,16^ focusing on two main aspects: team composition and interface design. The team includes multiple roles: physicians, designers, usability and UX experts, ML researchers and programmers. This is especially relevant since a multidisciplinary team can achieve a more complete understanding of the real requisites of the project and mitigate workflow disruptions.^11,23^ The interface was carefully designed, the layout was implemented focusing on usability and the colours used were extracted from a PC logo previously created by an artist As described in,^24^ the aesthetic part of the application has a direct effect on its perceived usability, so that an effort must be made to create a visually attractive application. The scores obtained in the UEQ-S hedonic category reflect the acceptability of the aesthetics, although none of the participants commented explicitly on the visual aspect.

The main strength of our work was that our method helped us to be aware of the different pitfalls that had been pointed out in previous works^2,9,10,11,12,13^ using the HCPs’ insights. The usability test showed that the system is *good enough* for the participants. However, specific changes are needed to fit the different contexts in which CDSS is deployed to maximize its usability. The system was thoroughly validated by means of the predictive models evaluated in a previous publication^29^ and the estimate of usability and UX in the present study. We created anecdotal evidence to support the UX dimension within the standard usability tests. We also managed to get a diverse sample of participants in terms of roles and countries, providing us with a richer version of health providers’ PC needs in different parts of the world.

However, our work also had some limitations: first, the model was evaluated by a small number of participants, and since ML models are deterministic once trained, the machine side was only assessed in six different cases, although this was not a problem with respect to the accuracy of the predictions since the models had already been evaluated. The second limitation is the requirement of the manual input of hospital admission data because it is disconnected from the EHR. This could be an advantage for primary care physicians working in rural areas but breaks the premise of automating the data collection as much as possible and increases the possibility of human errors. The heterogeneity of the participants’ roles made evaluating the platform's fit to specific conditions more difficult even though it helped us to find errors, it limited us to evaluating its use in specific phases of the clinical workflow. In addition, in this demo, we did not address some problems regarding the temporal variability related to different medical centres data distributions.^56^

In future work, we would like to adapt the tool to the different roles and clinical workflows we have identified. A further study focused on the different PC roles and their needs regarding Aleph PC would be needed to provide a perfect fit and improve usability. Further adaptations and validations of the ML models would be needed to ensure the models preserve their predictive power in other populations. We would also need to create a pilot for potential users to incorporate the tools into their daily routines and gather long-term feedback. Further research on ML explainability and reportability is needed to create a transparent and auditable system to improve the acceptance of the technology by helping to avoid legal problems.^11,53^ A study focused on mortality and frailty prediction accuracy by HCPs may be needed to estimate a fair baseline to improve the predictive models.

## Conclusions

Our main findings indicate that the predictive models performed better than the baseline composed of HCPs’ predictions. The system has good UX hedonic qualities, that is, the participants were interested in using the tool and they positively rated the fact of having helped to identify patients with bad prognoses and they did not feel their independence threatened by The Aleph PC. Performance regarding usability was modest but acceptable. Based on the notes obtained from the think-aloud method, the authors hypothesize that the usability scores for the current version are maximized and would only improve if the tool was adapted to the different roles and contexts represented in the participant's sample. We created anecdotal evidence that an iterative user-centred validation, including UX, provides a broader vision to address CDSS acceptance issues. Our objective is thus to advance further in including objective PC criteria.

## Supplemental Material

sj-xlsx-1-dhj-10.1177_20552076221150735 - Supplemental material for User-centred design of a clinical decision support system for palliative care: Insights from healthcare professionals Click here for additional data file. Supplemental material, sj-xlsx-1-dhj-10.1177_20552076221150735 for User-centred design of a clinical decision support system for palliative care: Insights from healthcare professionals by Vicent Blanes-Selva, Sabina Asensio-Cuesta, Ascensión Doñate-Martínez, Felipe Pereira Mesquita and Juan M. García-Gómez in Digital Health

## References

[1] Berner ES. Clinical decision support systems, Vol. 233. New York: Springer, 2007.

[2] Sutton RT Pincock D Baumgart DC, et al. An overview of clinical decision support systems: benefits, risks, and strategies for success. NPJ Digital Medicine 2020; 3: 1–10. 32047862 10.1038/s41746-020-0221-y PMC7005290

[3] Tundjungsari V Mudzakir Sofro AS Sabiq A, et al. Investigating clinical decision support systems success factors with usability testing. International Journal of Advanced Computer Science and Applications (IJACSA) 2017; 8: 548–554.

[4] Belard A Buchman T Forsberg J, et al. Precision diagnosis: a view of the clinical decision support systems (cdss) landscape through the lens of critical care. J Clin Monit Comput 2017; 31: 261–271. 26902081 10.1007/s10877-016-9849-1

[5] Yang Q Zimmerman J Steinfeld A, et al. Investigating the heart pump implant decision process: opportunities for decision support tools to help. In Proceedings of the 2016 CHI Conference on Human Factors in Computing Systems. (pp. 4477–4488), 2016. 10.1145/2858036.2858373 PMC5101017 27833397

[6] Devaraj S Viernes S. Barriers and facilitators to clinical decision support systems adoption: A systematic review. International Journal of Trends in Business Administration 2014; 2: 36–53.

[7] Elwyn G Scholl I Tietbohl C, , et al. “Many miles to go. . . ”: a systematic review of the implementation of patient decision support interventions into routine clinical practice. BMC Med Inform Decis Mak 2013; 13: 1–10. 24625083 10.1186/1472-6947-13-S2-S14 PMC4044318

[8] Yang Q Zimmerman J Steinfeld A. Review of medical decision support tools: Emerging opportunity for interaction design. IASDR 2015 Interplay Proceedings, 2015.

[9] Khairat S Marc D Crosby W, et al. Reasons for physicians not adopting clinical decision support systems: critical analysis. JMIR Med Inform 2018; 6: e24. 29669706 10.2196/medinform.8912 PMC5932331

[10] Liberati EG Ruggiero F Galuppo L, et al. What hinders the uptake of computerized decision support systems in hospitals? A qualitative study and framework for implementation. Implement Sci 2017; 12: 1–13. 28915822 10.1186/s13012-017-0644-2 PMC5602839

[11] Yu K-H Beam AL Kohane IS. Artificial intelligence in healthcare. Nature Biomedical Engineering 2018; 2: 719–731. 10.1038/s41551-018-0305-z 31015651

[12] Carroll C Marsden P Soden P, et al. Involving users in the design and usability evaluation of a clinical decision support system. Comput Methods Programs Biomed 2002; 69: 123–135. 12100792 10.1016/s0169-2607(02)00036-6

[13] Press A McCullagh L Khan S, et al. Usability testing of a complex clinical decision support tool in the emergency department: lessons learned. JMIR human Factors 2015; 2: e4537. 10.2196/humanfactors.4537 PMC4797671 27025540

[14] Genes N Kim MS Thum FL, et al. Usability evaluation of a clinical decision support system for geriatric ed pain treatment. Appl Clin Inform 2016; 7: 128–142. 27081412 10.4338/ACI-2015-08-RA-0108 PMC4817340

[15] Richardson S Mishuris R O’Connell A, et al. “Think aloud” and “near live” usability testing of two complex clinical decision support tools. Int J Med Inf 2017; 106: 1–8. 10.1016/j.ijmedinf.2017.06.003 PMC5679128 28870378

[16] Thum F Kim MS Genes N, et al. Usability improvement of a clinical decision support system. In International Conference of Design, User Experience, and Usability. (pp. 125–131). Springer, 2014.

[17] Li AC Kannry JL Kushniruk A, et al. Integrating usability testing and think-aloud protocol analysis with “near-live” clinical simulations in evaluating clinical decision support. Int J Med Inf 2012; 81: 761–772. 10.1016/j.ijmedinf.2012.02.009 22456088

[18] Eccles DW Arsal G. The think aloud method: what is it and how do i use it? Qualitative research in sport. Exercise and Health 2017; 9: 514–531.

[19] Brooke J, et al. Sus-a quick and dirty usability scale. Usability Evaluation in Industry 1996; 189: 4–7.

[20] Lewis JR. The system usability scale: past, present, and future. International Journal of Human–Computer Interaction 2018; 34: 577–590.

[21] Wallach DP Flohr LA Kaltenhauser A. Beyond the buzzwords: On the perspective of ai in ux and vice versa. In International Conference on Human-Computer Interaction. (pp. 146–166). Springer, 2020.

[22] Bevan N. What is the difference between the purpose of usability and user experience evaluation methods. In Proceedings of the Workshop UXEM, volume 9, (pp. 1–4). Citeseer, 2009.

[23] Mahadevaiah G Prasad RV Bermejo I, et al. Artificial intelligence-based clinical decision support in modern medical physics: selection, acceptance, commissioning, and quality assurance. Med Phys 2020; 47: e228–e235. 32418341 10.1002/mp.13562 PMC7318221

[24] Moran K. The aesthetic-usability effect, 2017. Aesthetic-usability effect, last accessed on 2 June 2022. https://www.nngroup.com/articles/.

[25] Tan A Durbin M Chung FR, et al. Design and implementation of a clinical decision support tool for primary palliative care for emergency medicine (PRIM-ER). BMC Med Inform Decis Mak 2020; 20: 1–11. 31992301 10.1186/s12911-020-1021-7 PMC6988238

[26] Maas EA Murray SA Engels Y, et al. What tools are available to identify patients with palliative care needs in primary care: a systematic literature review and survey of European practice. BMJ Support Palliat Care 2013; 3: 444–451. 10.1136/bmjspcare-2013-000527 24950525

[27] Pocock LV Wye L French L, et al. Barriers to GPs identifying patients at the end-of-life and discussions about their care: a qualitative study. Fam Pract 2019; 36: 639–643. 30649266 10.1093/fampra/cmy135

[28] Llop-Medina L Fu Y Garcés-Ferrer J, et al. Palliative care in older people with multimorbidities: a scoping review on the palliative care needs of patients, carers, and health professionals. Int J Environ Res Public Health 2022; 19: 3195. 35328881 10.3390/ijerph19063195 PMC8954932

[29] Blanes-Selva V Doñate-Martínez A Linklater G, et al. Complementary frailty and mortality prediction models on older patients as a tool for assessing palliative care needs. Health Informatics J 2022; 28: 14604582221092592. PMID: 35642719. 35642719 10.1177/14604582221092592

[30] Cai CJ Reif E Hegde N, et al. Human-centered tools for coping with imperfect algorithms during medical decision-making. In Proceedings of the 2019 chi conference on human factors in computing systems. (pp. 1–14), 2019.

[31] Mahoney FI, et al. Functional evaluation: the Barthel index. Md State Med J 1965; 14: 61–65. 14258950

[32] Charlson M Szatrowski TP Peterson J, et al. Validation of a combined comorbidity index. J Clin Epidemiol 1994; 47: 1245–1251. 7722560 10.1016/0895-4356(94)90129-5

[33] Lundberg SM Lee S-I. A unified approach to interpreting model predictions. In Proceedings of the 31st international conference on neural information processing systems. (pp. 4768–4777), 2017.

[34] Parker C Scott S Geddes A. Snowball sampling. London: Sage research methods foundations, 2019.

[35] Nielsen J. Usability engineering. San Diego, CA: Morgan Kaufmann, 1994.

[36] Wong HB Lim GH. Measures of diagnostic accuracy: sensitivity, specificity, PPV and NPV. Proceedings of Singapore Healthcare 2011; 20: 316–318.

[37] Schrepp M Hinderks A Thomaschewski J. Design and evaluation of a short version of the user experience questionnaire (ueq-s). International Journal of Interactive Multimedia and Artificial Intelligence 2017; 4: 103–108.

[38] Nielsen J. Why you only need to test with 5 users, 2000. https://www.nngroup.com/articles/why-you-only-need-to-test-with-5-users/, last accessed 2 June 2022.

[39] Bangor A Kortum P Miller J. Determining what individual sus scores mean: adding an adjective rating scale. J Usability Stud 2009; 4: 114–123.

[40] Downar J Goldman R Pinto R, et al. The “surprise question” for predicting death in seriously ill patients: a systematic review and meta-analysis. CMAJ 2017; 189: E484–E493. 28385893 10.1503/cmaj.160775 PMC5378508

[41] Yang Q Steinfeld A Zimmerman J. Unremarkable ai: Fitting intelligent decision support into critical, clinical decision-making processes. In Proceedings of the 2019 CHI Conference on Human Factors in Computing Systems (pp. 1–11), 2019.

[42] Melin-Johansson C Palmqvist R Rönnberg L. Clinical intuition in the nursing process and decision-making—a mixed-studies review. J Clin Nurs 2017; 26: 3936–3949. 28329439 10.1111/jocn.13814

[43] Esmaeilzadeh P Sambasivan M Kumar N, et al. Adoption of clinical decision support systems in a developing country: antecedents and outcomes of physician’s threat to perceived professional autonomy. Int J Med Inf 2015; 84: 548–560. 10.1016/j.ijmedinf.2015.03.007 25920928

[44] Laka M Milazzo A Merlin T. Factors that impact the adoption of clinical decision support systems (cdss) for antibiotic management. Int J Environ Res Public Health 1901; 18: 2021. 10.3390/ijerph18041901 PMC7920296 33669353

[45] Tolmie P Pycock J Diggins T, et al. Unremarkable computing. In *Proceedings of the SIGCHI conference on Human factors in computing systems* (pp. 399–406), 2002.

[46] Akhloufi H Verhaegh SJC Jaspers MWM, et al. A usability study to improve a clinical decision support system for the prescription of antibiotic drugs. PloS One 2019; 14: e0223073. 31553785 10.1371/journal.pone.0223073 PMC6760771

[47] Moore DC Payne S Van den Block L, et al. Strategies for the implementation of palliative care education and organizational interventions in long-term care facilities: a scoping review. Palliat Med 2020; 34: 558–570. 32009516 10.1177/0269216319893635 PMC7222696

[48] Ferrell BR Chung V Koczywas M, et al. Dissemination and implementation of palliative care in oncology. J Clin Oncol 2020; 38: 995. 32023151 10.1200/JCO.18.01766 PMC7082157

[49] Steigleder T Kollmar R Ostgathe C. Palliative care for stroke patients and their families: barriers for implementation. Front Neurol 2019; 10: 164. 30894836 10.3389/fneur.2019.00164 PMC6414790

[50] Fricker ZP Serper M. Current knowledge, barriers to implementation, and future directions in palliative care for end-stage liver disease. Liver Transpl 2019; 25: 787–796. 30758901 10.1002/lt.25434

[51] Finucane AM O’Donnell H Lugton J, et al. Digital health interventions in palliative care: a systematic meta-review. NPJ Digital Medicine 2021; 4: 1–10. 33824407 10.1038/s41746-021-00430-7 PMC8024379

[52] Zemplényi AT Csikós Á Csanádi M, et al. Implementation of palliative care consult service in Hungary–integration barriers and facilitators. BMC Palliat Care 2020; 19: 1–12. 32220251 10.1186/s12904-020-00541-0 PMC7102442

[53] Reyes M Meier R Pereira S, et al. On the interpretability of artificial intelligence in radiology: challenges and opportunities. Radiology: Artificial Intelligence 2020; 2: e190043. 32510054 10.1148/ryai.2020190043 PMC7259808

[54] Shortliffe EH Sepúlveda MJ. Clinical decision support in the era of artificial intelligence. Jama 2018; 320: 2199–2200. 30398550 10.1001/jama.2018.17163

[55] Ghassemi M Oakden-Rayner L Beam AL. The false hope of current approaches to explainable artificial intelligence in health care. The Lancet Digital Health 2021; 3: e745–e750. 34711379 10.1016/S2589-7500(21)00208-9

[56] Sáez C Gutiérrez-Sacristán A Kohane I, et al. Ehrtemporalvariability: delineating temporal data-set shifts in electronic health records. GigaScience 2020; 9: 1–7. 10.1093/gigascience/giaa079 PMC7391413 32729900

